# Editorial: Exploring links between social communication and mental health

**DOI:** 10.3389/fpsyt.2022.1130363

**Published:** 2023-01-10

**Authors:** Daniel Holzinger, Johannes Fellinger

**Affiliations:** ^1^Institute of Neurology of Senses and Language, Hospital of St. John of God, Linz, Austria; ^2^Research Institute for Developmental Medicine, Johannes Kepler University Linz, Linz, Austria; ^3^Institute of Linguistics, University of Graz, Graz, Austria; ^4^Division of Social Psychiatry, University Clinic for Psychiatry and Psychotherapy, Medical University of Vienna, Vienna, Austria

**Keywords:** social communication, pragmatic language, emotional and behavioral problems, peer-relationships, mental health

Social communication (SC) implies the selection of appropriate non-verbal and verbal messages and their appropriate interpretation within a social context. SC serves a rich variety of communicative functions of varying degrees of complexity (such as attracting somebody's attention, commenting, asking for information, sharing information or emotions and humor, arguing or negotiating). Essentially, SC includes the mastering of reciprocity and following the rules of human conversation (including turn-taking skills, topic adherence or communicative repair strategies). SC difficulties are highly heterogeneous and encompass clinical as well as non-clinical groups. Impairments of SC can be associated with mental, behavioral and neurodevelopmental disorders, and are a core symptom of autism spectrum disorders and the recently introduced ICD-11 category of “developmental language disorder with impairment of mainly pragmatic language.” In addition, SC difficulties can be associated with non-clinical populations such as children of low socio-economic background or with other experiences of deprivation. Finally, in case of insignificant impact on everyday functioning, SC difficulties can be regarded as an expression of human neurodiversity.

This Research Topic aimed to increase our understanding of SC skills in childhood and youth and the correlations with current or subsequent mental health problems. The collection of articles includes a systematic review by our working group (Dall et al.) and a number of empirical articles on the association between SC skills and child mental health (Fellinger et al.; Rimehaug and Kårstad) and the wellbeing of the family environment (Laister et al.). The relationships between early childhood experiences and child SC development are explored by a conceptual paper by Jethava et al. and a population-based empirical study by Law et al.. Finally, Weber et al. offer an instrument to measure SC skills in an adult population with special needs.

## Present state summary

The systematic review (Dall et al.) demonstrates an increasing number of publications on the associations between SC skills and mental health in children and young adults, with mostly satisfactory to high quality and a recently growing number of publications, particularly in Europe. Population-based studies find significantly higher rates of SC problems in children with all dimensions of mental health problems (externalizing, internalizing) and peer-relationship problems. Studies with a longitudinal design demonstrate effects of earlier SC skills on later mental health outcomes. Research examining the shapes of trajectories of symptoms of mental health problems (depression, ADHD, conduct problems) consistently shows higher rates of SC difficulties in the groups with early onset and persistent trajectories. Despite of associations between SC skills and other dimensions of child development (such as language, social development, executive functioning, intelligence) these dimensions are only found to explain a limited rate of the variance of SC skills so that SC must be regarded as a self-contained developmental domain.

## Effects of SC skills on mental health outcomes

The cross-sectional study by Fellinger et al. on a population of adults with deafness and intellectual disabilities shows that language and SC skills rather than intellectual and adaptive skills are significantly correlated with maladaptive behavior. Our results confirm findings on the impact of communication problems on challenging behavior in people with disorders of intellectual development.

The significant role of the acquisition of SC skills in pre-schoolers with autism spectrum disorder on their mothers' wellbeing is shown by the study by Laister et al. Increases in SC during early intervention are found to be the strongest negative predictor of post-intervention maternal stress exceeding gains in language, non-verbal cognition and adaptive skills. This study is remarkable, as there is highly limited research on the effects of child SC difficulties on the family environment.

In a population-based study on adolescents Rimehaug and Kårstad investigate associations between emotional vocabulary and SC and mental health. Whereas, SC skills [measured by the CCC-2; Bishop (1)] show significant negative correlations with mental health problems, emotional vocabulary is not associated with SC or mental health. As a consequence, the authors suggest that the expansion of emotional vocabulary not integrated in more general SC training might not improve mental health outcomes.

## Early experiences and SC

Drawing on longitudinal data from the Growing up in Scotland Study Law et al. investigate the relationships between home learning environment and parental mental health in the first years of life and structural and pragmatic language (SC) skills at the end of primary school. Parental mental health is found to be specifically related with SC whereas home learning environment is associated with structural and pragmatic language outcomes. In addition, a mediating role of socio-emotional adjustment at school entry between early parental mental health and later child SC and language difficulties is reported.

The conceptual paper by Jethava et al. offers a developmental neuropsychological framework that tries to explain the interplay between attachment and the child's development of SC, language, emotion and cognition, thus arguing for an integrated approach to assessments and interventions for SC.

## Measuring SC

The development and evaluation of easy-to-use instruments for assessing SC in different population is an essential requirement for the identification of risk factors for mental health problems, intervention planning and research. Weber et al. demonstrate first promising data on the validity and feasibility of a care-giver SC questionnaire for adults with intellectual disability independent of communication mode (whether spoken or signed).

## Conclusions and future directions

This Research Topic demonstrates the availability of international research providing consistent evidence for significant correlations between SC skills and mental health outcomes. SC difficulties co-occur with mental health problems, can trigger mental health problems and moderate their trajectories. These findings warrant the implementation and development of tools for the identification and monitoring of SC skills, preventive programs targeting parental mental health and home environment in the early years, and the integration of SC interventions in mental health and education programs.

## Author contributions

DH prepared a first version of the manuscript. JF reviewed the manuscript. All authors contributed to the article and approved the submitted version.
